# ELAV/Hu RNA-binding protein family: key regulators in neurological disorders, cancer, and other diseases

**DOI:** 10.1080/15476286.2025.2471133

**Published:** 2025-02-25

**Authors:** Huxitaer Wutikeli, Ting Xie, Wenjun Xiong, Yin Shen

**Affiliations:** a Eye Center, Renmin Hospital of Wuhan University, Wuhan University, Wuhan, Hubei, China; b Division of Life Science, The Hong Kong University of Science and Technology, Special Administrative Region (SAR), Kowloon, Hong Kong, China; c Department of Biomedical Sciences, City University of Hong Kong, Hong Kong, China; d Frontier Science Center for Immunology and Metabolism, Medical Research Institute, School of Medicine, Wuhan University, Wuhan, Hubei, China

**Keywords:** RNA-binding proteins (RBPs), ELAV/Hu, neurological disorders, cancer, neurodegeneration, inflammation, co-transcriptional and post-transcriptional regulation, mRNA stability, alternative splicing, disease biomarkers

## Abstract

The ELAV/Hu family represents a crucial group of RNA-binding proteins predominantly expressed in neurons, playing significant roles in mRNA transcription and translation. These proteins bind to AU-rich elements in transcripts to regulate the expression of cytokines, growth factors, and the development and maintenance of neurons. Elav-like RNA-binding proteins exhibit remarkable molecular weight conservation across different species, highlighting their evolutionary conservation. Although these proteins are widely expressed in the nervous system and other cell types, variations in the DNA sequences of the four Elav proteins contribute to their distinct roles in neurological disorders, cancer, and other Diseases . Elavl1, a ubiquitously expressed family member, is integral to processes such as cell growth, ageing, tumorigenesis, and inflammatory diseases. Elavl2, primarily expressed in the nervous and reproductive systems, is critical for central nervous system and retinal development; its dysregulation has been implicated in neurodevelopmental disorders such as autism. Both Elavl3 and Elavl4 are restricted to the nervous system and are involved in neuronal differentiation and excitability. Elavl3 is essential for cerebellar function and has been associated with epilepsy, while Elavl4 is linked to neurodegenerative diseases, including Parkinson’s and Alzheimer’s diseases. This paper provides a comprehensive review of the ELAV/Hu family’s role in nervous system development, neurological disorders, cancer, and other diseases.

## Introduction

**Figure 1.**
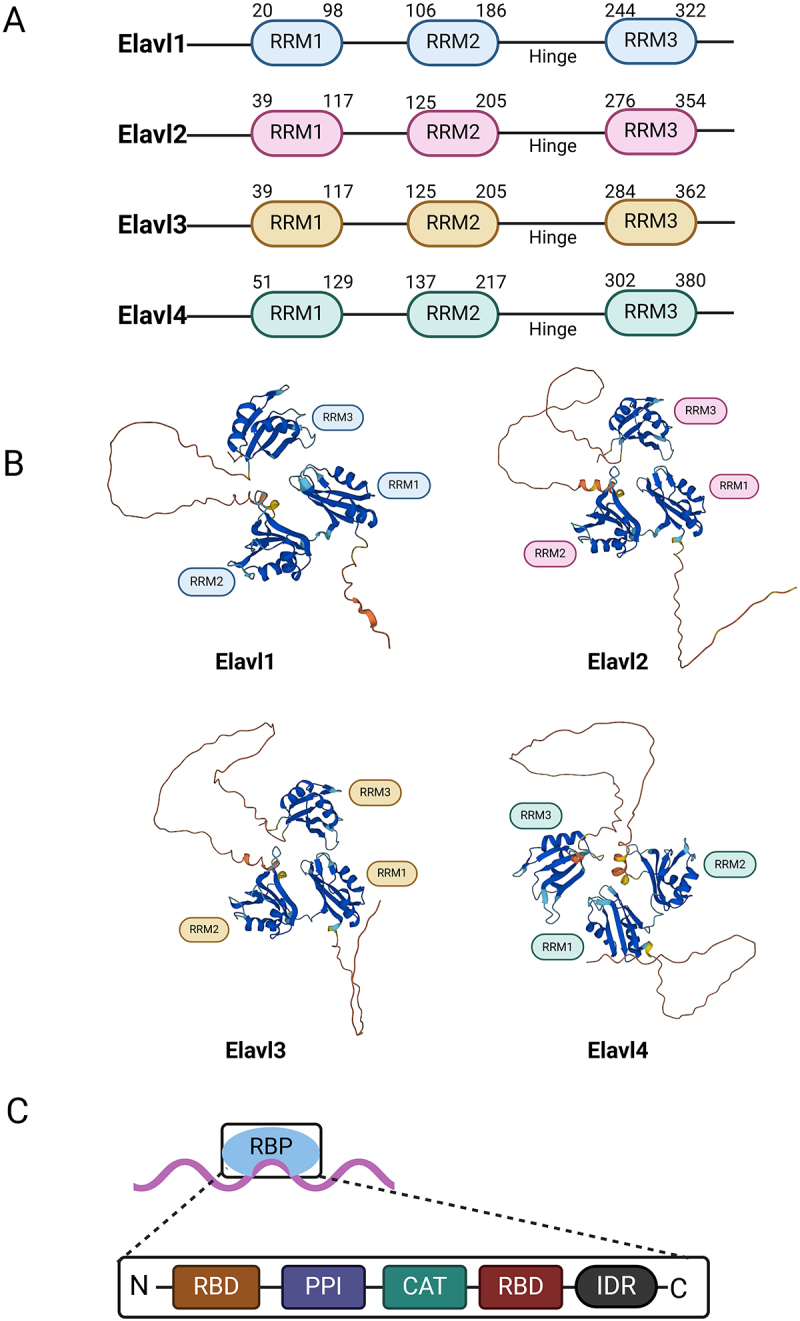
The structure of ELAV/Hu RNA-binding protein (A) A schematic of the four human ELAV proteins, including the three RNA recognition motifs (RRM) and hinge region. (B) AlphaFold predicted protein structures for Elavl1 and Elavl4. The general organization of the protein is the same with three core RRM domains and a disordered hinge domain. (C) General modular structure of RNA-binding proteins. The binding of multiple RNA-binding proteins (RBPs) onto the same target RNA dictates the metabolic fate of the transcript. RBPs display a modular architecture, as they may be endowed with several domains. RBPs bind to the specific transcript through one or more RNA-binding domains (RBDs). Protein-protein interaction (PPI) and catalytic (CAT) domains mediate the binding to other partner proteins and the activity of the RBPs, respectively. In addition, several RBPs contain intrinsically-disordered regions (IDRs), which generally lack a defined three-dimensional structure. The number and type of each domain varies greatly among RBPs. A large number and combination of post-translational modifications (PTMs) further increases the structural and functional complexity of RNA-binding proteins. Figure 1 created with BioRender.com.

**Figure 2.**
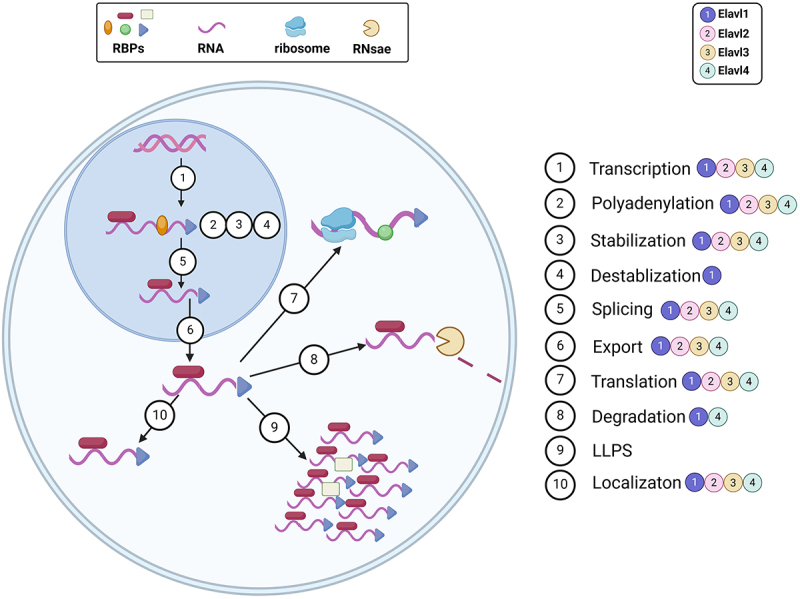
The function of ELAV/Hu RNA-binding protein RNA-binding proteins-mediated modulation of RNA metabolism. The schematic picture describes some of the processes through which RNA-binding proteins (RBPs) influence RNA biology inside the nucleus and the cytoplasm of a cell. These processes include (but are not limited to): transcription, polyadenylation, stabilization, RNA splicing, export, cellular localization, translation, degradation, and liquid-liquid phase separation (LLPS). Figure 2 created with BioRender.com.

**Figure 3.**
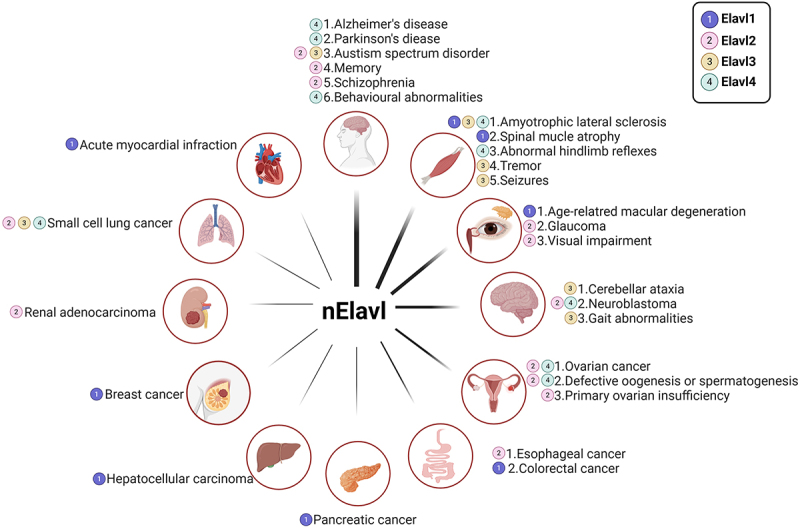
The relationship between ELAV/Hu RNA binding protein and disease. Diseases or pathophysiological processes related to ELAV proteins. Diseases related to ELAV proteins are included in the figure. Colors indicate which of the nELAV genes the phenotype or disease has been associated with Elavl1 (purple), Elavl2 (pink), Elavl3 (yellow), Elavl4 (green). Figure 3 created with BioRender.com.

Mammalian gene expression is a complex and tightly regulated process. Beyond transcription, mRNA processing, transport, stability, and translation are crucial for regulating gene expression in the nervous system. These post-transcriptional processes are regulated by the interaction of certain RNA-binding proteins (RBPs) with specific sequences in target mRNAs, while others are unconventional RBPs (ucRBPs) that lack known RNA-binding domains (RBDs) and often exhibit no sequence specificity [1]. (Figure 1C) RBPs, which bind to single- or double-stranded RNA, have selective affinity for various RNAs and regulate multiple RNA functions, influencing cell fate [2,3]. They protect target RNAs from pre-mRNA transcription in the nucleus through nuclear export until translation is completed in the cytoplasm [4]. Through numerous in vivo and in vitro studies, over 1,000 RBPs with distinct functions have been identified in humans and mice [5]. RBPs participate in processes including mRNA processing and maturation, coordination and stabilization of protein complexes, alternative splicing, polyadenylation, silencing, degradation of mature mRNA, as well as intracellular transport, localization, and stabilization [6,7]. (Figure 2) Given their involvement in numerous post-transcriptional mechanisms, RBP dysfunction may contribute to a variety of diseases, such as cardiovascular diseases, immune disorders, cancer, and neurodegenerative diseases [8,9]. Among them, RBPs are very closely linked to central nervous system diseases, including Alzheimer’s disease, Parkinson’s disease, autism, and schizophrenia [10–12]. (Figure 3)

In the nervous system, alternative splicing is highly prevalent, and RBPs help protect mRNA from premature translation and degradation during its transport from neurons to dendrites and axons [13]. One of the most extensively studied neuronal RBPs is the embryonic lethal abnormal visual system ELAV/Hu protein, which plays a key role in co-transcriptional regulation of pre-mRNA [14,15]. The protein family was first identified in *Drosophi*l*a* [16], where it encodes three neural-specific RBPs: Elav (Embryonic Lethal Abnormal Vision), Rbp9 (RNA-binding protein 9), and Fne (Found in neurons). These proteins are involved in neuron development and function in *Drosophi*l*a*, and mutations during embryonic development can cause embryonic lethality and visual defect [17]. ELAV/Hu proteins were later identified in humans as tumour-specific antigens in paraneoplastic neuropathy, particularly in lung cancer patients [18]. These antigens can trigger the production of anti-Hu antibodies, which cross the blood-brain barrier and cause neurological disorders characterized by dementia and spinal cord lesions [19]. Moreover, Hu proteins in humans show high homology to *Drosophi*l*a* Elav proteins. (Figure 1B) The four members of the Hu family – HuR, HuB, HuC, and HuD – correspond to Elavl1, Elavl2, Elavl3, and Elavl4 respectively. Elavl2 through Elavl4, also known as neuronal Elav proteins, are predominantly expressed in neurons, with high sequence homology (70–85%) among them [20–24]. In humans and mice, the ELAV family comprises four members – Elavl1 (HuR, ~36 kDa), Elavl2 (HuB, ~39 kDa), Elavl3 (HuC, ~39 kDa), and Elavl4 (HuD, ~42 kDa) [25]. In contrast, *Drosophila melanogaster* encodes a single Elav gene (~50 kDa) [26], while in *Caenorhabditis elegans* the homologs range from 40 to 50 kDa [27]. *Zebrafish* and *Xenopus laevis* exhibit Elav protein sizes similar to those in mammals (approximately 36–42 kDa) [28,29]. This remarkable conservation in molecular weight across species underscores the evolutionary importance of ELAV-like RNA-binding proteins [30]. Phylogenetic studies reveal that HuR, HuB, HuC, and HuD have followed distinct evolutionary trajectories, acquiring specialized functions in both neural and non-neural contexts. Moreover, comparative analyses in insects (e.g. Drosophila ELAV) and mammals support the notion that, following duplication, ancestral ELAV genes evolved tissue-specific regulatory elements that facilitated their functional specialization [31,32].

In mammals, ELAV/Hu proteins (Elavl1, Elavl2, Elavl3, and Elavl4) show dynamic localization, typically found in the nucleus or cytoplasm, depending on cell type, differentiation state, and external conditions. For example, HuR (Elavl1) is mainly nuclear but can move to the cytoplasm under stress, while HuD (Elavl4) is predominantly cytoplasmic, especially in neuronal axons and synapses. This flexible distribution highlights their diverse roles in mRNA regulation [33–36]. The Elav-like proteins contain three highly conserved RNA recognition motifs (RRM). (Figure 1A)The two tandem RRMs at the n-terminus are separated by a basic segment from the third RRMs and connected to the c-terminal polyadenylation recognition site through a variable sequence containing cis-acting elements for nuclear-cytoplasmic shuttling [37–39]. The polyadenylation site exhibits a strong affinity for the AU-rich elements (AREs) in the 3’-UTR of target mRNAs, where ELAV/Hu proteins bind to regulate the expression of cytokines, growth factors, and proteins involved in neuronal differentiation and maintenance [40–44]. Binding at this site stabilizes the Elavl-mRNA complex by preventing exonuclease-mediated degradation or by facilitating interactions with other RNA-stabilizing factors [23,45]. In addition, the third RRM mediates binding to the poly (A) tail of mRNA, which proves that Elav-like proteins can bind to both AU-rich elements and poly (A) tails [44,46]. Beyond their role in neuronal development and maintenance, the co-transcriptional regulatory functions of the ELAV/Hu protein family are increasingly linked to a growing number of diseases [47–50]. (Figure 3) A deeper understanding of these proteins will offer insights into their mechanisms in nervous system development, the pathogenesis of neurological disorders, cancer, and other diseases.

## The structure and function of Elavl1/HuR protein

Elavl1 was first successfully cloned and characterized in 1996 as an RNA-binding protein that is widely expressed in various tissues, including adipose tissue, spleen, intestines, and testes [41]. It plays a critical role in regulating cell proliferation and differentiation and is essential for normal embryonic development and survival. Studies in mouse models have demonstrated that *Elavl1* knockout leads to embryonic lethality during the second trimester, highlighting its necessity for normal embryonic survival and development [51]. Under physiological conditions, the *Elavl1* gene in mice produces three mRNA isoforms with different 3′ untranslated region (UTR) lengths. The most abundant form is the 2.4 kb mRNA, which is widely expressed, while the 1.5 kb mRNA is testis-specific and a 6.0 kb neuron-specific mRNA is expressed in the brain [52]. These isoforms can interconvert under different conditions to fulfil distinct functions [53–57]. *Elavl1* predominantly resides in the nucleus, where it regulates splicing [58–61] and polyadenylation [62]. Normally, AU-rich elements (AREs) destabilize mRNA by accelerating deadenylation, but Elavl1 competes with these elements, thereby increasing mRNA stability and translation [63,64]. Elavl1 is also involved in various physiological processes, including cellular senescence [65], tumour growth [36], stress responses and apoptosis [66], neuroinflammation [67], and motor neuron injury [68]. By regulating target mRNA, Elavl1 influences the cellular response to different stimuli, such as stress, proliferation, differentiation, apoptosis, ageing, immunity and inflammation [40,69,70]. In the central nervous system, Elavl1 not only supports normal neural development but is also closely associated with neuroinflammation and motor neuron injury [71]. Elavl1 is implicated in the activation of astrocytes and microglia, and is particularly overexpressed in the spinal cord microglia of humans and mice with amyotrophic lateral sclerosis (ALS) [72]. In neuron-specific *Elavl1* knockout models, increased expression of activated caspase-3 in motor neurons leads to motor neuron injury [73–77].

In addition to its role in the nervous system, Elavl1 also plays a crucial role in inflammation and cancer. In the cytoplasm, Elavl1 plays a key role in cell and tissue differentiation by regulating mRNAs involved in inflammation and regeneration [67,69]. Inflammatory cytokines, such as interleukin-6, interleukin-8, transforming growth factor-β, C-reactive protein (CRP), tumour necrosis factor-α, and complement components, are stabilized and expressed in various immune cells, including fibroblasts, macrophages, and T cells, through Elavl1-mediated regulation [78–81]. In inflammatory diseases such as vasculitis, Elavl1 promotes the inflammatory response [68]. Moreover, inhibition of *Elavl1* significantly alleviates motor dysfunction and hyperalgesia in models of autoimmune encephalomyelitis. Elavl1 also plays a role in chronic inflammatory diseases, such as pancreatitis [82], rheumatoid arthritis [83] and asthma [84]. Elavl1 is the most extensively studied member of the Elav protein family in cancer research. It is expressed in various cancers, including lung cancer, neuroblastoma, small cell lung cancer, prostate cancer, and ovarian cancer [36], and it promotes tumour formation, cell proliferation, and metastasis. Recent studies have shown that Elavl1 interacts with *IDH1* to regulate metabolic activity in pancreatic ductal adenocarcinoma [85]. Evidence suggests that *Elavl1* acts as an oncogene, enhancing the transcriptional activity of target mRNAs and promoting cancer hallmarks, such as sustained proliferative signalling, evasion of growth suppression, invasion and metastasis, induction of angiogenesis, replicative immortality, inhibition of cell death, and promotion of tumour-related inflammation and immune evasion [86–93]. The expression of *Elavl1* is an important prognostic marker for benign and malignant tumours. For example, in ductal breast carcinoma, increased cytoplasmic *Elavl1* is associated with poor differentiation, larger tumour size, lower survival rates, and resistance to radiotherapy and chemotherapy [94,95]. In meningioma, Elavl1 serves as a prognostic marker. Currently, targeting *Elavl1* is considered a promising therapeutic strategy in cancer treatment. Silencing *Elavl1* is being explored as an adjunct target for chemotherapy in pancreatic cancer [96]. Additionally, IL-10 reduces the expression of inflammatory cytokines post-transcriptionally by inhibiting *Elavl1* expression. Inhibition of *Elavl1* has also been shown to enhance the sensitivity of breast and colon cancer cells to low-dose radiotherapy by approximately two-fold [95].

In the visual system, *Elavl1* moves from the nucleus to the cytoplasm in response to oxidative stress, particularly in the human trabecular meshwork, where it regulates intraocular pressure. This process is linked to glaucoma by controlling stress-response proteins like Hsp70 and p53 [97,98]. Reduced cytoplasmic Elavl1 in glaucoma models and human samples leads to lower levels of these proteins, contributing to glaucoma development. Elavl1 also plays a role in diabetic retinopathy by stabilizing mRNAs of TNF-α and VEGF, promoting disease progression [99]. In age-related macular degeneration (AMD), Elavl1 worsens retinal pigment epithelial cell dysfunction, leading to drusen formation. Inhibiting *Elavl1* expression has shown promise in slowing diabetic retinopathy, making it a potential therapeutic target for retinal diseases [99].

## Neuronal ELAVs (neuronal Elav-like proteins, nELAVs) protein structure and function

The nElavls family of proteins consists of three members: Elavl*2*, Elavl3, and Elavl4, which serve as molecular markers specific to neurons [100]. These proteins bind to target mRNAs, regulating processes such as RNA nuclear export, alternative splicing, polyadenylation, and translation, playing critical roles in neuronal development and maintenance [14]. Research in zebrafish demonstrates that nElavls are expressed in early-generated neurons within the retina, such as amacrine cells (ACs) and retinal ganglion cells (RGCs). Additionally, nELAVs expression has been observed in post-mitotic fibroblasts of the lens in rats and mice [101]. Increasing evidence suggests that nELAVs are highly enriched in neurons and play significant roles in neurodegenerative diseases, including fragile X syndrome [102], Parkinson’s disease [103], Huntington’s disease [104], and Alzheimer’s disease [105,106]. nELAVs promote neuronal development by facilitating central nervous system plasticity, and supporting peripheral nerve regeneration. They also enhance axonal growth, and nerve recovery following injury [20,24,40,49,107,108]. Borgonetti et al. [40] Studies indicate that *Elavl2*, *Elavl3*, and *Elavl4* in the mammalian cerebral cortex exhibit distinct temporal and spatial expression patterns, suggesting specialized roles in neuronal development beyond their shared functions, with *Elavl2* and *Elavl4* displaying similar patterns that differ slightly from *Elavl3* [109,110].

### The structure and function of Elavl2 protein

Elavl*2* protein was initially detected in neuronal progenitor cells of the developing embryonic cortical layer, followed by the expression of Elavl4 and Elavl3 proteins. It was specifically expressed in the progenitor cells of the mouse telencephalon, with expression levels increasing rapidly from embryonic day E16.5. At the same time, the neuronal progenitor cell marker Tbr2 was co-expressed with Elavl2, suggesting that Elavl*2* may play a role in the differentiation of neuronal progenitor cells in the brain [111]. Additionally, Elavl2 is closely associated with the 3′UTR of *Foxg1*, promoting its expression by counteracting the inhibitory effect of miR-9 and thereby regulating the differentiation of embryonic forebrain progenitor cells [112]. Previous studies revealed that *Elavl2* begins to express in the mouse retina at embryonic day E12.5 and continues long after birth, specifically in amacrine cells (ACs), retinal ganglion cells (RGCs), and horizontal cells. In an *Elavl2*-knockout mouse model, Elavl2 was found to play a critical role in the generation and differentiation of retinal ACs. *Elavl2* deficiency led to the down-regulation of transcription factors Nr4a2, Barhl2, and Neurod1, reducing the production of GABAergic and glycinergic transmitters in ACs and ultimately impairing visual function and acuity. Moreover, Elavl2 interacts with GABAB receptors at both RNA and protein levels, participating in signal transduction [109,113] ELAVl2 has also been implicated in neurological disorders, including schizophrenia and autism. Genome-wide association studies (GWAS) have identified *ELAVl2* as a key gene in schizophrenia development through family screenings and genotyping [114]. Whole-genome sequencing has also linked *ELAVl2* to autism spectrum disorders [49]. Regionally, Elavl2 exhibits cell-specific expression patterns in the hippocampus, primarily localized to CA3 pyramidal neurons and hilar interneurons. In a kainic acid (KA)-induced epilepsy model, Elavl2 protein expression was significantly reduced in CA3 neurons, along with reduced mRNA levels of the downstream factor GAP-43. These results suggest that Elavl2 is regulated by the KAKAR signalling axis and plays a role in activity-dependent RNA regulation [115]. Interestingly, Elavl2 is the only Elav protein expressed in honeybees, where alternative splicing produces over 40 protein isoforms involved in the brain’s mushroom bodies, which are centres for olfactory learning and memory. These regulatory mechanisms may be further influenced by nuclear localization via microexons within the variable hinge region of *Elavl2* [116].

Beyond neurons, *Elavl2* has been found to be highly conserved and enriched in human spermatogonia and mouse gonads, where it is primarily localized in the nucleus and is essential for the proliferation and survival of spermatogonia. Elavl2 plays a crucial role in the post-transcriptional regulation of spermatogonial stem cell maintenance and differentiation. During mouse development, *Elavl2* expression begins in testicular gonadal cells at E13.5 and remains elevated during testicular development, but decreases rapidly following spermatogonial stem cell differentiation. A similar pattern is observed in human testicular development. In vitro studies have shown that Elavl2 promotes spermatogonia proliferation and inhibits apoptosis by activating the ERK and AKT signalling pathways and increasing the expression of c-Fos and Myc proteins [117]. In female mice ovaries, Elavl2 is involved in the regulation of P-body mRNA, which is associated with RNA decay and cytoplasmic RNP granule storage. Elavl2 also promotes the assembly of P-body granules by enhancing DDX6 translation in oocytes, a process essential for primordial follicle formation [118].

### The structure and function of Elavl3 protein

Elavl3 protein is highly expressed in the mature cerebral cortex and shows a unique expression pattern compared to Elavl3 and Elavl4. Specifically, Elavl3 is expressed in Purkinje cells and the hippocampus of the cerebellum, indicating its potential role in regulating cerebellar cell differentiation and maintaining cerebellar structure and function [119]. This hypothesis was confirmed using an *Elavl3* whole-body knockout model, where the deletion of *Elavl3* had no apparent effects on the overall health, vitality, or lifespan of the mice. However, Elavl3 plays a crucial role in maintaining the morphology and axonogenesis of cerebellar Purkinje cells. It regulates the polarity of Purkinje neurons by mediating specific splicing events in G protein-coupled receptors in embryonic exons. Loss of *Elavl3* leads to Purkinje cell swelling, formation of spherical cell bodies, and axonal degeneration, disrupting synaptic connections with the cerebellar nuclei, which causes severe cerebellar ataxia and progressive motor dysfunction. The absence of *Elavl3* also decreases neuronal excitability, impairing motor function. Ince-Dunn et al. utilized HITS-CLIP technology to demonstrate that Elavl3 binds to U-rich regions in glutamine synthetase mRNA, regulating the glutamate synthesis pathway and promoting normal neuronal electrical activity [120]. The proposed mechanism suggests that *Elavl3* deficiency results in impaired selective splicing of glutaminase RNA, leading to imbalances in the glutamate network, potentially increasing susceptibility to epilepsy. Indeed, haploinsufficiency or heterozygous mutations in *Elavl3* have been linked to spontaneous epilepsy. In *Elavl3* knockout mice, symptoms of epilepsy include transient clonic convulsive seizures and high-frequency neuronal electrical activity [120]. Additionally, *Elavl3* loss affects selective polyadenylation, producing different 3′UTR lengths in target genes and leading to the downregulation of neural markers Gad1 and TubβIII, which delays the differentiation of GABAergic neurons [121]. Elavl3 plays a key role in the development and functioning of nerve cells, particularly in the formation and maintenance of glutamatergic excitatory neurons. Studying the role of Elavl3 offers new insights into the pathogenesis and potential treatments for neuronal axonal degeneration.

### The structure and function of Elavl4 protein

The gene encoding *Elavl4* is located on chromosome 1p34 in humans and 49.5 cM on chromosome 4 in mice. The mouse *Elavl4* gene spans approximately 146 kb and contains seven exons (exon 2 to exon 8) that span about 44 kb of DNA [122]. Among nElavls, Elavl4 was the first member to be discovered and successfully replicated, and it remains the most extensively studied. Studies have shown that *Elavl4* mRNA can be detected as early as E10.5 in the embryonic brains of mice and rats, peaking at E16.5 and gradually decreasing postnatally [123]. Elavl4 binds to around 1304 mRNAs, including those encoding mTORC1-responsive ribosomal proteins and translation factors. Our previous research demonstrated that Elavl4 binds to Satb1 to regulate Neurod1, promoting the differentiation of retinal progenitors and amacrine cells [109]. Beyond its role in neuronal proliferation and differentiation, Elavl4 also influences neurite growth. Mutations in the *Elavl4* gene during early embryonic development lead to transient abnormalities in the hypoglossal and glossopharyngeal nerves, as well as reduced neural progenitor proliferation. In *Elavl4-*deficient mice, abnormal hindlimb reflexes are observed, though no significant neurodegeneration occurs [124]. In the adult brain’s subventricular zone (SVZ), Elavl4 stabilizes the mRNA of adenine-thymidine (AT)-rich SATB1, promoting the differentiation of neural stem/progenitor cells into neurons. Knockout mice lacking *Elavl4* exhibit an increase in neural progenitor cells but reduced early neuronal differentiation, suggesting that Elavl4 regulates multiple stages of neurodevelopment [125]. Specifically, Elavl4 appears to negatively regulate neural progenitor cell proliferation [24], facilitating the exit of neuronal precursor cells from the cell cycle and promoting the differentiation of postmitotic neurons [126]. While much of the research on Elavl4 focuses on its role in neurogenesis, it also plays an essential part in neuronal function, survival, learning, memory, and synaptic plasticity after injury [120,127]. In developing neurons, Elavl4 is localized in axon growth cones, and in mature neurons, it is found in axons and dendrites, where it promotes neurite growth. The protein regulates the stability and expression of target mRNA products, including brain-derived neurotrophic factor (BDNF), GAP-43, Tau, acetylcholinesterase (AChE), c-Fos, and N-myc [128,129]. Elavl4 promotes axonal formation, microtubule assembly, and growth cone development, playing a neuroprotective role by enhancing axonal growth and contributing to synaptic plasticity mechanisms [130]. In the dorsal root ganglion (DRG), Elavl4 promotes axonal regeneration after injury. Silencing *Elavl4* leads to decreased axonal regeneration, and its loss disrupts dendrite formation in pyramidal neurons and reduces motor neuron axons [131]. These findings indicate that Elavl4 plays a critical role in the dendrite formation of specific neuronal subtypes.

Elavl4 is also expressed in glutamatergic neurons in the hippocampus and neocortex, where it regulates neuronal excitability in neural circuits. It has been implicated in neurodegenerative diseases such as Parkinson’s disease (PD) and Alzheimer’s disease (AD), as well as motor neuron diseases such as spinal muscular atrophy (SMA) and amyotrophic lateral sclerosis (ALS). Multiple studies have shown that genetic variations in ELAVL4 are closely linked to the age at onset of Parkinson’s disease (PD). Replication studies, such as GenePD, have confirmed these associations [132], and additional research has localized ELAVL4 to the PARK10 locus – suggesting that its variability may influence PD risk [133]. Notably, kinases like LRRK2 (commonly mutated in familial PD) can phosphorylate HuD, a modification that may alter its RNA-binding properties and indirectly lead to abnormal expression of multiple downstream genes, thereby contributing to PD pathology [47,103]. Moreover, changes in HuD expression disrupt the mRNA networks essential for maintaining synaptic structure and function, accelerating the degeneration of midbrain dopaminergic neurons and further driving PD progression [47]. Additionally, Elavl4 binds to the long non-coding RNA BACE1-AS, stabilizing it and further enhancing *BACE*1 production, thus contributing to Aβ formation [134]. In motor neurons, Elavl4 interacts with survival motor neuron (SMN) protein, influencing target mRNA levels and axon and dendrite development. A deficiency in *Elavl4* or reduced SMN protein levels can result in defects in motor neuron development and synapse formation, leading to spinal muscular atrophy [135]. Moreover, Elavl4 stabilizes the long 3′UTR variant of *SOD*1 mRNA, suggesting that it contributes to the pathogenesis of ALS through post-transcriptional regulation of *SOD*1 and by influencing oxidative stress. Thus, Elavl4 May represent a novel biomarker or therapeutic target for treating ALS [127]. In summary, Elavl4 binds to target mRNAs to promote neuronal maturation and function, and it may contribute to neurodegenerative diseases through its regulation of mRNA stability.

## Summary and prospect

The RNA-binding protein ELAV/Hu family, which includes four key members (Elavl1, Elavl2, Elavl3, and Elavl4), plays critical roles in post-transcriptional gene regulation. While Elavl1 (HuR) is broadly expressed in various tissues and regulates cell proliferation, differentiation, and stress responses, its roles in organismal growth, ageing, tumorigenesis, and oxidative stress have been extensively characterized. In contrast, the other three members – collectively referred to as neuronal ELAVs (nELAVs) – are predominantly expressed in the nervous system, where they are essential for neuron-specific RNA regulation. Recent evidence suggests that the duplication events generating the four ELAV family members are relatively recent and consistent with the ‘out of testis’ hypothesis, as several of these genes exhibit high expression in reproductive tissues, implying that testis-specific factors may have driven their emergence. Future studies integrating gene age estimation tools (e.g. phylostratigraphy) and single-cell transcriptomics of human gonads may clarify whether ELAV family members represent evolutionarily young genes co-opted for reproductive functions [136].

The evolutionary conservation of ELAV/Hu proteins across species offers a unique perspective on functional evolution. In *Drosophila*, for example, ELAV/Hu proteins regulate neuronal mRNA alternative polyadenylation (APA) through overlapping activities that shape the 3′ UTR landscape critical for neurodevelopment [137]. Similarly, research in plants has shown that rice ELAV/Hu homologs (e.g. EHL1) directly bind to MADS-box transcripts to regulate floral organ development, suggesting that these proteins may have played an ancient role in developmental patterning that predates the divergence of animals and plants [138]. Future comparative studies of structural motifs, such as the RRM domains, across different kingdoms could reveal conserved regulatory principles alongside lineage-specific innovations.

Beyond neuronal maintenance, emerging evidence highlights a role for ELAV/Hu proteins in cancer biology. Elavl4 is considered a potential oncogene in glioblastoma because it stabilizes pro-survival mRNAs, while HuR (Elavl1) promotes tumour progression in hepatocellular carcinoma and gastric cancer, with its high cytoplasmic expression linked to poor prognosis [139]. Inhibitors such as CMLD-2, targeting HuR-RNA interactions, are in preclinical testing and may lead to therapies against nELAVs [140]. Moreover, there is a provocative possibility that nELAVs might be exploited to modulate tumour immunogenicity through their effects on the stability of immune checkpoint gene transcripts. In addition to their established roles in neuronal processes, ELAV/Hu proteins display remarkable versatility. In humans, nELAVs have been detected in germ cells where they may help safeguard meiosis-specific transcripts, indicating a broader functional spectrum than previously thought. Systemic dysregulation of HuR in metabolic diseases such as non-alcoholic steatohepatitis further hints at potential crosstalk between RNA-binding proteins and metabolic signalling – a frontier that remains to be fully explored.

Looking towards the future, several promising research directions emerge. Synthetic biology applications, such as engineering nELAVs with programmable RNA-binding domains (e.g. CRISPR-Cas13 fusion proteins), could allow for precise control of transcript stability in gene therapy. Advancements in dynamic RNA imaging, including technologies like vLUME VR, may enable real-time visualization of nELAV-RNA interactions within three-dimensional neuronal networks. Preliminary evidence that HuR modulates circadian clock genes raises the question of whether nELAVs integrate RNA metabolism with circadian rhythms, particularly in the context of neurodegeneration. Furthermore, exploring plant – animal hybrid systems – using rice EHL1 mutants to determine if animal ELAV proteins can rescue developmental defects – could provide valuable insights into the evolutionary conservation and divergence of these regulatory proteins.

In summary, the Elav/Hu family exemplifies how RNA-binding proteins bridge molecular specificity with systemic adaptability. While current research has illuminated their crucial roles in neurodevelopment and cancer, emerging tools – from spatial omics to RNA-targeted therapeutics – promise to unravel their complete mechanistic spectrum. By embracing cross-disciplinary approaches in evolutionary biology, synthetic immunology, and beyond, future studies could transform these proteins from mere molecular players into foundational pillars of precision medicine.

## Data Availability

I confirm I understand the terms of the share upon reasonable request data policy. I confirm I have included a Data Availability Statement in my manuscript Data sharing is not applicable to this article as no new data were created or analysed in this study.

## References

[1] Ray D, Laverty KU, Jolma A, et al. RNA-binding proteins that lack canonical RNA-binding domains are rarely sequence-specific. Sci Rep. 2023;13(1):10. doi: 10.1038/s41598-023-32245-9 37002329 PMC10066285

[2] Hentze MW, Castello A, Schwarzl T, et al. A brave new world of RNA-binding proteins. Nat Rev Mol Cell Biol. 2018;19(5):327–341. doi: 10.1038/nrm.2017.130 29339797

[3] Xiao R, Chen JY, Liang Z, et al. Pervasive chromatin-RNA binding protein interactions enable RNA-Based regulation of transcription. Cell. 2019;178(1):107–121.e118. doi: 10.1016/j.cell.2019.06.001 31251911 PMC6760001

[4] Glisovic T, Bachorik JL, Yong J, et al. RNA-binding proteins and post-transcriptional gene regulation. FEBS Lett. 2008;582(14):1977–1986. doi: 10.1016/j.febslet.2008.03.004 18342629 PMC2858862

[5] Gerstberger S, Hafner M, Tuschl T. A census of human RNA-binding proteins. Nat Rev Genet. 2014;15(12):829–845. doi: 10.1038/nrg3813 25365966 PMC11148870

[6] Alami NH, Smith RB, Carrasco MA, et al. Axonal transport of TDP-43 mRNA granules is impaired by ALS-causing mutations. Neuron. 2014;81(3):536–543. doi: 10.1016/j.neuron.2013.12.018 24507191 PMC3939050

[7] Díaz-Muñoz MD, Turner M. Uncovering the role of RNA-Binding proteins in gene expression in the immune system. Front Immunol. 2018;9:1094. doi: 10.3389/fimmu.2018.01094 29875770 PMC5974052

[8] de Bruin RG, Rabelink TJ, van Zonneveld AJ, et al. Emerging roles for RNA-binding proteins as effectors and regulators of cardiovascular disease. Eur Heart J. 2017;38:1380–1388. doi: 10.1093/eurheartj/ehw567 28064149

[9] Guo J, Qu H, Chen Y, et al. The role of RNA-binding protein tristetraprolin in cancer and immunity. Med Oncol. (Northwood, London, England). 2017;34, 196 doi: 10.1007/s12032-017-1055-6 29124478

[10] Kinoshita C, Kubota N, Aoyama K. Interplay of RNA-Binding proteins and microRNAs in neurodegenerative diseases. Int J Mol Sci. 2021;22(10):5292. doi: 10.3390/ijms22105292 34069857 PMC8157344

[11] Gantois I, Khoutorsky A, Popic J, et al. Metformin ameliorates core deficits in a mouse model of fragile X syndrome. Nat Med. 2017;23(6):674–677. doi: 10.1038/nm.4335 28504725

[12] Gumina V, Colombrita C, Fallini C, et al. TDP-43 and NOVA-1 RNA-binding proteins as competitive splicing regulators of the schizophrenia-associated TNIK gene. Biochim et Biophys Acta Gene Regul mechanisms. 2019;1862(9):194413. doi: 10.1016/j.bbagrm.2019.194413 PMC7818381 31382054

[13] Du X, Xiao R, Wu Z. An emerging role of chromatin-interacting RNA-binding proteins in transcription regulation. Essays Biochem. 2020;64(6):907–918. doi: 10.1042/ebc20200004 33034346

[14] Hilgers V. Regulation of neuronal RNA signatures by ELAV /hu proteins. RNA. 2023;14(2):e1733. doi: 10.1002/wrna.1733 35429136

[15] Wei L, Lai EC. Regulation of the alternative neural transcriptome by ELAV/Hu RNA binding proteins. Front Genet. 2022;13:848626. doi: 10.3389/fgene.2022.848626 35281806 PMC8904962

[16] Robinow S, Campos AR, Yao KM, et al. The elav gene product of drosophila, required in neurons, has three RNP consensus motifs. Science. 1988;242(4885):1570–1572. doi: 10.1126/science.3144044 3144044

[17] Yao KM, Samson ML, Reeves R, et al. Gene elav of drosophila melanogaster: a prototype for neuronal-specific RNA binding protein gene family that is conserved in flies and humans. J Neurobiol. 1993;24(6):723–739. doi: 10.1002/neu.480240604 8331337

[18] Keene JD. Why is Hu where? Shuttling of early-response-gene messenger RNA subsets. Proceedings of the National Academy of Sciences of the United States of America; 1999. 96:5–7. doi: 10.1073/pnas.96.1.5 PMC33538 9874760

[19] Graus F, Dalmau J. Paraneoplastic neurological syndromes: diagnosis and treatment. Curr Opin Neurol. 2007;20(6):732–737. doi: 10.1097/WCO.0b013e3282f189dc 17992098

[20] Pascale A, Amadio M, Scapagnini G, et al. Neuronal ELAV proteins enhance mRNA stability by a PKCalpha-dependent pathway. Proceedings of the National Academy of Sciences of the United States of America; 2005. 102:12065–12070. doi: 10.1073/pnas.0504702102 PMC1189326 16099831

[21] Mirisis AA, Kopec AM, Carew TJ. ELAV proteins bind and stabilize C/EBP mRNA in the induction of long-term memory in Aplysia. J Neurosci. 2021;41(5):947–959. doi: 10.1523/jneurosci.2284-20.2020 33298536 PMC7880277

[22] Peng SS, Chen CY, Xu N, et al. RNA stabilization by the au-rich element binding protein, HuR, an ELAV protein. Embo J. 1998;17(12):3461–3470. doi: 10.1093/emboj/17.12.3461 9628881 PMC1170682

[23] Ford LP, Watson J, Keene JD, et al. ELAV proteins stabilize deadenylated intermediates in a novel in vitro mRNA deadenylation/degradation system. Genes & Devel. 1999;13(2):188–201. doi: 10.1101/gad.13.2.188 9925643 PMC316394

[24] Ratti A, Fallini C, Cova L, et al. A role for the ELAV RNA-binding proteins in neural stem cells: stabilization of Msi1 mRNA. J Cell Sci. 2006;119(7):1442–1452. doi: 10.1242/jcs.02852 16554442

[25] Good PJ. A conserved family of elav-like genes in vertebrates. Proceedings of the National Academy of Sciences of the United States of America; 1995. 92:4557–4561. doi: 10.1073/pnas.92.10.4557 PMC41983 7753842

[26] Lisbin MJ, Qiu J, White K. The neuron-specific RNA-binding protein ELAV regulates neuroglian alternative splicing in neurons and binds directly to its pre-mRNA. Genes Dev. 2001;15(19):2546–2561. doi: 10.1101/gad.903101 11581160 PMC312793

[27] Fujita M, Kawano T, Ohta A, et al. Neuronal expression of a Caenorhabditis elegans elav-like gene and the effect of its ectopic expression. Biochem Biophys Res Commun. 1999;260(3):646–652. doi: 10.1006/bbrc.1999.0957 10403820

[28] Ogawa Y, Kakumoto K, Yoshida T, et al. Elavl3 is essential for the maintenance of Purkinje neuron axons. Sci Rep. 2018;8(1):2722. doi: 10.1038/s41598-018-21130-5 29426875 PMC5807307

[29] He ZX, Song HF, Liu TY, et al. HuR in the medial prefrontal cortex is critical for stress-induced synaptic dysfunction and depressive-like symptoms in mice. Cerebral Cortex (New York, N.Y). 1991;29(6):2737–2747. doi: 10.1093/cercor/bhz036 30843060

[30] Good PJ. The role of elav-like genes, a conserved family encoding RNA-binding proteins, in growth and development. Semin Cell Dev Biol. 1997;8(6):577–584. doi: 10.1006/scdb.1997.0183 9642172

[31] Guo F, Yan L, Guo H, et al. The transcriptome and DNA methylome landscapes of human primordial germ cells. Cell. 2015;161(6):1437–1452. doi: 10.1016/j.cell.2015.05.015 26046443

[32] 刘莹. (家蚕Elav基因的克隆分析和时空表达研究 [Doctoral dissertation]. 苏州大学; 2007.

[33] Colombrita C, Silani V, Ratti A. ELAV proteins along evolution: back to the nucleus? Mol Cell Neurosci. 2013;56:447–455. doi: 10.1016/j.mcn.2013.02.003 23439364

[34] Brennan CM, Steitz JA. HuR and mRNA stability. CMLS Cell Mol Life Sci. 2001;58(2):266–277. doi: 10.1007/PL00000854 11289308 PMC11146503

[35] Hinman MN, Lou H. Diverse molecular functions of hu proteins. Cellular and molecular life sciences: CMLS. Cellular Mol Life Sci. 2008;65(20):3168–3181. doi: 10.1007/s00018-008-8252-6 18581050 PMC2580827

[36] Abdelmohsen K, Gorospe M. Posttranscriptional regulation of cancer traits by HuR. Wiley interdisciplinary reviews. RNA. 2010;1(2):214–229. doi: 10.1002/wrna.4 21935886 PMC3808850

[37] Fan XC, Steitz JA. HNS, a nuclear-cytoplasmic shuttling sequence in HuR. Proceedings of the National Academy of Sciences of the United States of America; 1998. 95:15293–15298. doi: 10.1073/pnas.95.26.15293 PMC28036 9860962

[38] King PH, Levine TD, Fremeau RT Jr., et al. Mammalian homologs of drosophila ELAV localized to a neuronal subset can bind in vitro to the 3’ UTR of mRNA encoding the id transcriptional repressor. J Neurosci. 1994;14(4):1943–1952. doi: 10.1523/jneurosci.14-04-01943.1994 8158249 PMC6577146

[39] Sakai K, Gofuku M, Kitagawa Y, et al. A hippocampal protein associated with paraneoplastic neurologic syndrome and small cell lung carcinoma. Biochem Biophys Res Commun. 1994;199(3):1200–1208. doi: 10.1006/bbrc.1994.1358 7511893

[40] Borgonetti V, Coppi E, Galeotti N. Targeting the RNA-Binding protein HuR as potential thera-peutic approach for neurological disorders: focus on Amyo-trophic lateral sclerosis (ALS), spinal muscle atrophy (SMA) and multiple sclerosis. Int J Mol Sci. 2021;22(19):10394. doi: 10.3390/ijms221910394 34638733 PMC8508990

[41] Ma WJ, Cheng S, Campbell C, et al. Cloning and characterization of HuR, a ubiquitously expressed Elav-like protein. J Biol Chem. 1996;271(14):8144–8151. doi: 10.1074/jbc.271.14.8144 8626503

[42] Liu J, Dalmau J, Szabo A, et al. Paraneoplastic encephalomyelitis antigens bind to the AU-rich elements of mRNA. Neurology. 1995;45(3):544–550. doi:10.1212/wnl.45.3.544 7898713

[43] Levine TD, Gao F, King PH, et al. Hel-N1: an autoimmune RNA-binding protein with specificity for 3’ uridylate-rich untranslated regions of growth factor mRNAs. Mol Cell Biol. 1993;13(6):3494–3504. doi: 10.1128/mcb.13.6.3494-3504.1993 8497264 PMC359819

[44] Ma WJ, Chung S, Furneaux H. The Elav-like proteins bind to au-rich elements and to the poly(a) tail of mRNA. Nucleic Acids Res. 1997;25(18):3564–3569. doi:10.1093/nar/25.18.3564 9278474 PMC146929

[45] Myer VE, Fan XC, Steitz JA. Identification of HuR as a protein implicated in auuua-mediated mRNA decay. Embo J. 1997;16(8):2130–2139. doi: 10.1093/emboj/16.8.2130 9155038 PMC1169815

[46] Chung S, Eckrich M, Perrone-Bizzozero N, et al. The Elav-like proteins Bind to a conserved regulatory element in the 3′-untranslated region of GAP-43 mRNA. J Biol Chem. 1997;272(10):6593–6598. doi: 10.1074/jbc.272.10.6593 9045688

[47] Silvestri B, Mochi M, Garone MG, et al. Emerging roles for the RNA-Binding protein HuD (ELAVL4) in nervous system diseases. Int J Mol Sci. 2022;23(23):14606. doi: 10.3390/ijms232314606 36498933 PMC9736382

[48] Mulligan MR, Bicknell LS. The molecular genetics of nELAVL in brain development and disease. Eur J Hum Genet. 2023;31(11):1209–1217. doi: 10.1038/s41431-023-01456-z 37697079 PMC10620143

[49] Berto S, Usui N, Konopka G, et al. ELAVL2-regulated transcriptional and splicing networks in human neurons link neurodevelopment and autism. Hum Mol Genet. 2016;25:2451–2464. doi: 10.1093/hmg/ddw110 27260404 PMC6086562

[50] Mirisis AA, Carew TJ. The ELAV family of RNA-binding proteins in synaptic plasticity and long-term memory. Neurobiol Learn Mem. 2019;161:143–148. doi: 10.1016/j.nlm.2019.04.007 30998973 PMC6529270

[51] Chi MN, Auriol J, Jégou B, et al. The RNA-binding protein ELAVL1/HuR is essential for mouse spermatogenesis, acting both at meiotic and postmeiotic stages. Mol Biol Cell. 2011;22(16):2875–2885. doi: 10.1091/mbc.E11-03-0212 21737689 PMC3154883

[52] Mansfield KD, Keene JD. Neuron-specific ELAV/Hu proteins suppress HuR mRNA during neuronal differentiation by alternative polyadenylation. Nucleic Acids Res. 2012;40(6):2734–2746. doi: 10.1093/nar/gkr1114 22139917 PMC3315332

[53] Hilgers V, Lemke SB, Levine M. ELAV mediates 3′ UTR extension in the drosophila nervous system. Genes Dev. 2012;26(20):2259–2264. doi: 10.1101/gad.199653.112 23019123 PMC3475798

[54] Rothamel K, Arcos S, Kim B, et al. ELAVL1 primarily couples mRNA stability with the 3′ UTRs of interferon-stimulated genes. Cell Rep. 2021;35(8):109178. doi: 10.1016/j.celrep.2021.109178 34038724 PMC8225249

[55] ELAVL1 gene (gene ID: 1994). National Center for Biotechnology Information (NCBI); [cited 2023 Oct 10. (2023ed].

[56] Liu S, Jiang X, Cui X, et al. Smooth muscle-specific HuR knockout induces defective autophagy and atherosclerosis. Cell Death Dis. 2021;12(4):385. doi: 10.1038/s41419-021-03671-2 33837179 PMC8035143

[57] Thierry-Mieg D, T MJ. A comprehensive cDNA-supported gene and transcripts annotation for Elavl1 (Mus musculus elav-like RNA binding protein 1). In: NCBI Gene Database. [cited 2025 Jan 29]. ed. 2006.

[58] Gauchotte G, Hergalant S, Vigouroux C, et al. Cytoplasmic overexpression of RNA-binding protein HuR is a marker of poor prognosis in meningioma, and HuR knockdown decreases meningioma cell growth and resistance to hypoxia. J Pathol. 2017;242(4):421–434. doi: 10.1002/path.4916 28493484

[59] Akaike Y, Masuda K, Kuwano Y, et al. HuR regulates alternative splicing of the TRA2β gene in human colon cancer cells under oxidative stress. Mol Cell Biol. 2014;34(15):2857–2873. doi: 10.1128/mcb.00333-14 24865968 PMC4135568

[60] Chang SH, Elemento O, Zhang J, et al. ELAVL1 regulates alternative splicing of eIF4E transporter to promote postnatal angiogenesis. Proceedings of the National Academy of Sciences of the United States of America; 2014. 111:18309–18314. doi: 10.1073/pnas.1412172111 PMC4280608 25422430

[61] Briata P, Chen CY, Giovarelli M, et al. HuR regulates alternative splicing by mediating the nuclear export of splicing factors. Mol Cell. 2005;20:81–93.

[62] Dutertre M, Chakrama FZ, Combe E, et al. A recently evolved class of alternative 3′-terminal exons involved in cell cycle regulation by topoisomerase inhibitors. Nat Commun. 2014;5(1):3395. doi: 10.1038/ncomms4395 24577238

[63] Zarei M, Lal S, Parker SJ, et al. Posttranscriptional upregulation of IDH1 by HuR establishes a powerful survival phenotype in pancreatic cancer cells. Cancer Res. 2017;77(16):4460–4471. doi: 10.1158/0008-5472.Can-17-0015 28652247 PMC5922269

[64] Blanco FF, Jimbo M, Wulfkuhle J, et al. The mRNA-binding protein HuR promotes hypoxia-induced chemoresistance through posttranscriptional regulation of the proto-oncogene PIM1 in pancreatic cancer cells. Oncogene. 2016;35(19):2529–2541. doi: 10.1038/onc.2015.325 26387536 PMC6818728

[65] Wang W, Furneaux H, Cheng H, et al. HuR regulates p21 mRNA stabilization by UV light. Mol Cell Biol. 2000;20(3):760–769. doi: 10.1128/mcb.20.3.760-769.2000 10629032 PMC85192

[66] Zhang X, Zou T, Rao JN, et al. Stabilization of XIAP mRNA through the RNA binding protein HuR regulated by cellular polyamines. Nucleic Acids Res. 2009;37(22):7623–7637. doi: 10.1093/nar/gkp755 19825980 PMC2794158

[67] Filippova N, Nabors LB. ELAVL1 role in cell fusion and tunneling membrane nanotube formations with implication to treat glioma heterogeneity. Cancers (Basel). 2020;12(10):3069. doi: 10.3390/cancers12103069 33096700 PMC7590168

[68] Borgonetti V, Sanna MD, Lucarini L, et al. Targeting the RNA-Binding protein HuR alleviates neuroinflammation in experimental autoimmune encephalomyelitis: potential therapy for multiple sclerosis. Neurotherapeutics. 2021;18(1):412–429. doi: 10.1007/s13311-020-00958-8 33200288 PMC8116432

[69] Gherzi R, Trabucchi M, Ponassi M, et al. Akt2-mediated phosphorylation of Pitx2 controls Ccnd1 mRNA decay during muscle cell differentiation. Cell Death Differ. 2010;17(6):975–983. doi: 10.1038/cdd.2009.194 20019746

[70] Kang MJ, Ryu BK, Lee MG, et al. Nf-κB activates transcription of the RNA-Binding factor HuR, via PI3K-AKT signaling, to promote gastric tumorigenesis. Gastroenterology. 2008;135(6):2030–2042, e2031–2033. doi:10.1053/j.gastro.2008.08.009 18824170

[71] Skliris A, Papadaki O, Kafasla P, et al. Neuroprotection requires the functions of the RNA-binding protein HuR. Cell Death Differ. 2015;22(5):703–718. doi: 10.1038/cdd.2014.158 25301069 PMC4392069

[72] Shih RH, Wang CY, Yang CM. Nf-kappaB signaling pathways in neurological inflammation: a mini review. Front Mol Neurosci. 2015;8:77. doi: 10.3389/fnmol.2015.00077 26733801 PMC4683208

[73] Sun K, Li X, Chen X, et al. Neuron-specific HuR-deficient mice spontaneously develop motor neuron disease. J Immunol, (Baltim Md. : 1950) 2018;201(1):157–166. doi: 10.4049/jimmunol.1701501 29760195 PMC6008238

[74] Lu L, Zheng L, Si Y, et al. Hu antigen R (HuR) is a positive regulator of the RNA-binding proteins TDP-43 and FUS/TLS: implications for amyotrophic lateral sclerosis. J Biol Chem. 2014;289(46):31792–31804. doi: 10.1074/jbc.M114.573246 25239623 PMC4231657

[75] Kim H, Woo JH, Lee JH, et al. 22(R)-hydroxycholesterol induces HuR-dependent MAP kinase phosphatase-1 expression via mGlur5-mediated Ca2+/PKCα signaling. Biochim Biophys Acta. 2016;1859(8):1056–1070. doi: 10.1016/j.bbagrm.2016.05.008 27206966

[76] Chellappan R, Guha A, Si Y, et al. SRI-42127, a novel small molecule inhibitor of the RNA regulator HuR, potently attenuates glial activation in a model of lipopolysaccharide-induced neuroinflammation. Glia. 2022;70(1):155–172. doi: 10.1002/glia.24094 34533864 PMC8595840

[77] Garone MG, Birsa N, Rosito M, et al. Als-related FUS mutations alter axon growth in motoneurons and affect HuD/ELAVL4 and FMRP activity. Commun Biol. 2021;4(1):1025. doi: 10.1038/s42003-021-02538-8 34471224 PMC8410767

[78] Jehung JP, Kitamura T, Yanagawa-Matsuda A, et al. Adenovirus infection induces HuR relocalization to facilitate virus replication. Biochem Biophys Res Commun. 2018;495(2):1795–1800. doi: 10.1016/j.bbrc.2017.12.036 29225167

[79] Matsye P, Zheng L, Si Y, et al. HuR promotes the molecular signature and phenotype of activated microglia: implications for amyotrophic lateral sclerosis and other neurodegenerative diseases. Glia. 2017;65(6):945–963. doi: 10.1002/glia.23137 28300326 PMC7944581

[80] Shin JS, Choi HE, Seo S, et al. Berberine decreased inducible nitric oxide synthase mRNA stability through negative regulation of human antigen R in lipopolysaccharide-induced macrophages. J Pharmacol Exp Ther. 2016;358(1):3–13. doi: 10.1124/jpet.115.231043 27189969

[81] Kim Y, Noren Hooten N, Dluzen DF, et al. Posttranscriptional regulation of the inflammatory marker C-reactive protein by the RNA-binding protein HuR and MicroRNA 637. Mol Cell Biol. 2015;35(24):4212–4221. doi: 10.1128/mcb.00645-15 26438598 PMC4648813

[82] Peng W, Furuuchi N, Aslanukova L, et al. Elevated HuR in pancreas promotes a pancreatitis-like inflammatory microenvironment that facilitates tumor development. Mol Cell Biol. 2018;38(3):10 .1128/mcb.00427–17. doi: 10.1128/MCB.00427-17 PMC5770537 29133460

[83] Sugihara M, Tsutsumi A, Suzuki E, et al. Effects of infliximab therapy on gene expression levels of tumor necrosis factor α, tristetraprolin, T cell intracellular antigen 1, and hu antigen R in patients with rheumatoid arthritis. Arthritis and rheumatism. 2007;56(7):2160–2169. doi: 10.1002/art.22724 17599736

[84] Atasoy U, Curry SL, López de Silanes I, et al. Regulation of eotaxin gene expression by tnf-α and IL-4 through mRNA stabilization: involvement of the RNA-Binding protein HuR. J Immunol (Baltim, Md. : 1950) 2003;171(8):4369–4378. doi: 10.4049/jimmunol.171.8.4369 14530362

[85] Zarei M, Lal S, Vaziri-Gohar A, et al. RNA-Binding protein HuR regulates both mutant and wild-type IDH1 in IDH1-mutated cancer. Mol Cancer Res. 2019;17(2):508–520. doi: 10.1158/1541-7786.Mcr-18-0557 30266754 PMC6359963

[86] Li Z, Wang Y, Hu R, et al. LncRNA B4GALT1-AS1 recruits HuR to promote osteosarcoma cells stemness and migration via enhancing YAP transcriptional activity. Cell Prolif. 2018;51(6):e12504. doi: 10.1111/cpr.12504 30182452 PMC6528912

[87] Holmes B, Benavides-Serrato A, Freeman RS, et al. mTorc2/akt/hsf1/huR constitute a feed-forward loop regulating Rictor expression and tumor growth in glioblastoma. Oncogene. 2018;37(6):732–743. doi: 10.1038/onc.2017.360 29059166 PMC5805585

[88] Zhang Z, Huang A, Zhang A, et al. HuR promotes breast cancer cell proliferation and survival via binding to CDK3 mRNA. Biomed Pharmacother. 2017;91:788–795. doi: 10.1016/j.biopha.2017.04.063 28501005

[89] Balkhi MY, Iwenofu OH, Bakkar N, et al. Acts as a decoy in sarcomas to protect the tumor suppressor A20 mRNA from degradation by HuR. Sci Signal. 2013;6, ra63. 286):miR–29. doi: 10.1126/scisignal.2004177 PMC3885907 23901138

[90] Tang H, Wang H, Cheng X, et al. HuR regulates telomerase activity through TERC methylation. Nat Commun. 2018;9(1):2213. doi: 10.1038/s41467-018-04617-7 29880812 PMC5992219

[91] Osera C, Martindale JL, Amadio M, et al. Induction of VEGFA mRNA translation by CoCl2 mediated by HuR. RNA Biol. 2015;12(10):1121–1130. doi: 10.1080/15476286.2015.1085276 26325091 PMC4829335

[92] Schultz CW, Preet R, Dhir T, et al. Understanding and targeting the disease-related RNA binding protein human antigen R (HuR). Wiley interdisciplinary reviews. RNA. 2020;11(3):e1581. doi: 10.1002/wrna.1581 31970930 PMC7482136

[93] Brauß TF, Winslow S, Lampe S, et al. The RNA-binding protein HuR inhibits expression of CCL5 and limits recruitment of macrophages into tumors. Mol Carcinog. 2017;56(12):2620–2629. doi:10.1002/mc.22706 28731284

[94] Denkert C, Weichert W, Winzer KJ, et al. Expression of the elav-like protein HuR is associated with higher tumor grade and increased cyclooxygenase-2 expression in human breast carcinoma. Clin Cancer Res. 2004;10(16):5580–5586. doi: 10.1158/1078-0432.Ccr-04-0070 15328200

[95] Badawi A, Hehlgans S, Pfeilschifter J, et al. Silencing of the mRNA-binding protein HuR increases the sensitivity of colorectal cancer cells to ionizing radiation through upregulation of caspase-2. Cancer Lett. 2017;393:103–112. doi: 10.1016/j.canlet.2017.02.010 28219770

[96] Jakstaite A, Maziukiene A, Silkuniene G, et al. HuR mediated post-transcriptional regulation as a new potential adjuvant therapeutic target in chemotherapy for pancreatic cancer. World J Gastroenterol. 2015;21(46):13004–13019. doi: 10.3748/wjg.v21.i46.13004 26675757 PMC4674719

[97] Mochizuki H, Murphy CJ, Brandt JD, et al. Altered stability of mRNAs associated with glaucoma progression in human trabecular meshwork cells following oxidative stress. Invest Ophthalmol Vis Sci. 2012;53(4):1734–1741. doi: 10.1167/iovs.12-7938 22395891 PMC3342790

[98] Smedowski A, Liu X, Podracka L, et al. Increased intraocular pressure alters the cellular distribution of HuR protein in retinal ganglion cells – a possible sign of endogenous neuroprotection failure. Biochim Et Biophys Acta Mol Basis Disease 1864. 2018;1864(1):296–306. doi: 10.1016/j.bbadis.2017.10.030 29107807

[99] Amadio M, Pascale A, Cupri S, et al. Nanosystems based on siRNA silencing HuR expression counteract diabetic retinopathy in rat. Pharmacol Res. 2016;111:713–720. doi: 10.1016/j.phrs.2016.07.042 27475885

[100] Scheckel C, Drapeau E, Frias MA, et al. Regulatory consequences of neuronal elav-like protein binding to coding and non-coding RNAs in human brain. Elife. 2016;5. doi: 10.7554/eLife.10421 PMC4798961 26894958

[101] Link BA, Fadool JM, Malicki J, et al. The zebrafish young mutation acts non-cell-autonomously to uncouple differentiation from specification for all retinal cells. Development (Cambridge, England). 2000;127(10):2177–2188. doi: 10.1242/dev.127.10.2177 10769241

[102] Tiruchinapalli DM, Caron MG, Keene JD. Activity-dependent expression of ELAV/Hu RBPs and neuronal mRNAs in seizure and cocaine brain. J Neurochem. 2008;107(6):1529–1543. doi: 10.1111/j.1471-4159.2008.05718.x 19014379

[103] Noureddine MA, Qin XJ, Oliveira SA, et al. Association between the neuron-specific RNA-binding protein ELAVL4 and Parkinson disease. Hum Genet. 2005;117(1):27–33. doi: 10.1007/s00439-005-1259-2 15827745

[104] Zhao Q, Li C, Yu M, et al. HuR stabilizes HTT mRNA via interacting with its exon 11 in a mutant htt-dependent manner. RNA Biol. 2020;17(4):500–516. doi: 10.1080/15476286.2020.1712894 31928144 PMC7237150

[105] Subhadra B, Schaller K, Seeds NW. Neuroserpin up-regulation in the Alzheimer’s disease brain is associated with elevated thyroid hormone receptor-β1 and HuD expression. Neurochem Int. 2013;63(5):476–481. doi: 10.1016/j.neuint.2013.08.010 24036060 PMC3902180

[106] van der Linden RJ, Gerritsen JS, Liao M, et al. RNA-binding protein ELAVL4/HuD ameliorates Alzheimer’s disease-related molecular changes in human iPSC-derived neurons. Prog Neurobiol. 2022;217:102316. doi: 10.1016/j.pneurobio.2022.102316 35843356 PMC9912016

[107] Allen M, Bird C, Feng W, et al. HuD promotes BDNF expression in brain neurons via selective stabilization of the BDNF long 3′UTR mRNA. PLOS ONE. 2013;8(1):e55718. doi: 10.1371/journal.pone.0055718 23383270 PMC3561324

[108] Diaz-Garcia S, Ko VI, Vazquez-Sanchez S, et al. Nuclear depletion of RNA-binding protein ELAVL3 (HuC) in sporadic and familial amyotrophic lateral sclerosis. Acta Neuropathol. 2021;142(6):985–1001. doi: 10.1007/s00401-021-02374-4 34618203 PMC8568872

[109] Wutikeli H, Yu Y, Zhang T, et al. Role of Elavl-like RNA-binding protein in retinal development and signal transduction. Biochim Et Biophys Acta Mol Basis Disease 1871. 2024;1871(1):167518. doi: 10.1016/j.bbadis.2024.167518 39307290

[110] Yano M, Okano HJ, Okano H. Involvement of Hu and heterogeneous nuclear ribonucleoprotein K in neuronal differentiation through p21 mRNA post-transcriptional regulation. J Biol Chem. 2005;280(13):12690–12699. doi: 10.1074/jbc.M411119200 15671036

[111] Yano M, Hayakawa-Yano Y, Okano H. RNA regulation went wrong in neurodevelopmental disorders: the example of Msi/Elavl RNA binding proteins. Intl J Devlp Neurosci. 2016;55(1):124–130. doi: 10.1016/j.ijdevneu.2016.01.002 26796049

[112] Shibata M, Nakao H, Kiyonari H, et al. MicroRNA-9 regulates neurogenesis in mouse telencephalon by targeting multiple transcription factors. J Neurosci. 2011;31(9):3407–3422. doi: 10.1523/jneurosci.5085-10.2011 21368052 PMC6623912

[113] Wu M, Deng Q, Lei X, et al. Elavl2 regulates retinal function via modulating the differentiation of amacrine cells subtype. Invest Ophthalmol Vis Sci. 2021;62(7):1. doi:10.1167/iovs.62.7.1 PMC8185395 34061953

[114] Yamada K, Iwayama Y, Hattori E, et al. Genome-wide association study of schizophrenia in Japanese population. PLOS ONE. 2011;6(6):e20468. doi: 10.1371/journal.pone.0020468 21674006 PMC3108953

[115] Ohtsuka T, Yano M, Okano H. Acute reduction of neuronal RNA binding Elavl2 protein and Gap43 mRNA in mouse hippocampus after kainic acid treatment. Biochem Biophys Res Commun. 2015;466(1):46–51. doi: 10.1016/j.bbrc.2015.08.103 26325429

[116] Ustaoglu P, Gill JK, Doubovetzky N, et al. Dynamically expressed single ELAV/Hu orthologue elavl2 of bees is required for learning and memory. Commun Biol. 2021;4(1):1234. doi: 10.1038/s42003-021-02763-1 34711922 PMC8553928

[117] Yang C, Yao C, Ji Z, et al. RNA-binding protein ELAVL2 plays post-transcriptional roles in the regulation of spermatogonia proliferation and apoptosis. Cell Prolif. 2021;54(9):e13098. doi: 10.1111/cpr.13098 34296486 PMC8450129

[118] Kato Y, Iwamori T, Ninomiya Y, et al. ELAVL2-directed RNA regulatory network drives the formation of quiescent primordial follicles. EMBO Rep. 2019;20(12):e48251. doi: 10.15252/embr.201948251 31657143 PMC6893360

[119] Ogawa Y, Kakumoto K, Yoshida T, et al. Elavl3 is essential for the maintenance of purkinje neuron axons. Sci Rep. 2018;8(1):2722. doi: 10.1038/s41598-018-21130-5 29426875 PMC5807307

[120] Ince-Dunn G, Okano HJ, Jensen KB, et al. Neuronal elav-like (hu) proteins regulate RNA splicing and abundance to control glutamate levels and neuronal excitability. Neuron. 2012;75(6):1067–1080. doi: 10.1016/j.neuron.2012.07.009 22998874 PMC3517991

[121] Grassi E, Santoro R, Umbach A, et al. Choice of alternative polyadenylation sites, mediated by the RNA-Binding protein Elavl3 Plays a Role In Differentiation Of Inhibitory Neuronal Progenitors. Front Cellular Neurosci. 2018;12:518. doi: 10.3389/fncel.2018.00518 PMC6338052 30687010

[122] Inman MV, Levy S, Mock BA, et al. Gene organization and chromosome location of the neural-specific RNA binding protein Elavl4. Gene. 1998;208(2):139–145. 10. doi: 10.1016/S0378-1119(97)00615-X 9524251

[123] Hambardzumyan D, Sergent-Tanguy S, Thinard R, et al. AUF1 and Hu proteins in the developing rat brain: implication in the proliferation and differentiation of neural progenitors. J Neurosci Res. 2009;87(6):1296–1309. doi: 10.1002/jnr.21957 19115409

[124] Akamatsu W, Fujihara H, Mitsuhashi T, et al. The RNA-binding protein HuD regulates neuronal cell identity and maturation. Proceedings of the National Academy of Sciences of the United States of America; 2005. 102:4625–4630. doi: 10.1073/pnas.0407523102 PMC555491 15764704

[125] Wang F, Tidei JJ, Polich ED, et al. Positive feedback between RNA-binding protein HuD and transcription factor SATB1 promotes neurogenesis. Proceedings of the National Academy of Sciences of the United States of America; 2015. 112:E4995–5004. doi: 10.1073/pnas.1513780112 PMC4568658 26305964

[126] Joseph B, Orlian M, Furneaux H. p21waf1 mRNA contains a conserved element in its 3′-untranslated region that is bound by the Elav-like mRNA-stabilizing proteins. J Biol Chem. 1998;273(32):20511–20516. doi: 10.1074/jbc.273.32.20511 9685407

[127] Jung M, Lee EK. RNA–binding protein HuD as a versatile factor in neuronal and non–neuronal systems. Biology (Basel). 2021;10(5):361. doi: 10.3390/biology10050361 33922479 PMC8145660

[128] Perrone-Bizzozero N, Bolognani F. Role of HuD and other RNA-binding proteins in neural development and plasticity. J Neurosci Res. 2002;68(2):121–126. doi: 10.1002/jnr.10175 11948657

[129] Bronicki LM, Jasmin BJ. Emerging complexity of the HuD/ELAVl4 gene; implications for neuronal development, function, and dysfunction. RNA. 2013;19(8):1019–1037. doi: 10.1261/rna.039164.113 23861535 PMC3708524

[130] Sanna MD, Ghelardini C, Galeotti N. HuD-mediated distinct BDNF regulatory pathways promote regeneration after nerve injury. Brain Res. 2017;1659:55–63. doi: 10.1016/j.brainres.2017.01.019 28111162

[131] DeBoer EM, Azevedo R, Vega TA, et al. Prenatal deletion of the RNA-binding protein HuD disrupts postnatal cortical circuit maturation and behavior. J Neurosci. 2014;34(10):3674–3686. doi: 10.1523/jneurosci.3703-13.2014 24599466 PMC3942583

[132] DeStefano AL, Latourelle J, Lew MF, et al. Replication of association between ELAVL4 and Parkinson disease: the GenePD study. Hum Genet. 2008;124(1):95–99. doi: 10.1007/s00439-008-0526-4 18587682 PMC2716559

[133] Haugarvoll K, Toft M, Ross OA, et al. ELAVL4, PARK10, and the celts. Mov Disord. 2007;22(4):585–587. doi: 10.1002/mds.21336 17230446

[134] Kang MJ, Abdelmohsen K, Hutchison ER, et al. HuD regulates coding and noncoding RNA to induce APP→Aβ processing. Cell Rep. 2014;7(5):1401–1409. doi: 10.1016/j.celrep.2014.04.050 24857657 PMC4074355

[135] le H, Duy T, An PQ, et al. HuD and the survival motor neuron protein interact in Motoneurons and are essential for motoneuron development, function, and mRNA regulation. J Neurosci. 2017;37(48):11559–11571. doi: 10.1523/jneurosci.1528-17.2017 29061699 PMC5707763

[136] Li L, Dong J, Yan L, et al. Single-cell RNA-Seq analysis maps development of human germline cells and gonadal niche interactions. Cell Stem Cell. 2017;20(6):891–892. doi: 10.1016/j.stem.2017.05.009 28575695

[137] Wei L, Lee S, Majumdar S, et al. Overlapping activities of ELAV/Hu family RNA binding proteins specify the extended neuronal 3′ UTR landscape in Drosophila. Mol Cell. 2020;80(1):140–155.e146. doi: 10.1016/j.molcel.2020.09.007 33007254 PMC7546445

[138] 林学磊 (2021). 《水稻中ELAV/Hu类RNA结合蛋白的功能特征和作用机理研究》.

[139] 湛钊.孙达权(2021). HuR通过调控STRA6信号通路促进肝癌细胞的增殖,迁移及侵袭. [J]. 贵州医科大学, 2021

[140] 张小风,彭佑共,谢飞,裴芝皆, 李晓晖. RNA 结合蛋白 HuR 在胃癌中的表达和临床意义 [J]. 中国继续医学教育, 2019;11 (24):66–69.

